# COVID-19 and Mental Health: Psychological Impact of the Pandemic on Women during Pregnancy and Puerperium

**DOI:** 10.1192/j.eurpsy.2023.1359

**Published:** 2023-07-19

**Authors:** M. Cerioli, R. Cafaro, F. Giorgetti, A. Colombo, B. M. Dell’Osso

**Affiliations:** 1 Department of Biomedical and Clinical Sciences “Luigi Sacco”, University of Milan, Milan; 2 Department of Psychiatry, ASST Fatebenefratelli-Sacco, Milano, Italy; 3 Department of Psychiatry and Behavioral Sciences, Stanford University, Stanford, CA, United States; 4 CRC “Aldo Ravelli” for Neurotechnology and Experimental Brain Therapeutics, University of Milan, Milan, Italy

## Abstract

**Introduction:**

The COVID-19 pandemic has taken a great toll on world population in the last 2 years across different groups. Women in pregnancy and in the perinatal period are especially vulnerable to sources of stress, with repercussions on their own mental health and on the wellness of the newborn.

**Objectives:**

We aimed at assessing the impact of COVID-19 related stress on women’s psychosocial status and evaluate the potential development of anxiety or depressive symptoms in our cohort.

**Methods:**

From March 2020 to November 2020, our hospital was appointed as an HUB for treating pregnant women positive for Sars-CoV-2 infection. Sociodemographic and clinical data were collected, and patients were delivered different self-report psychometric tests such as the GHQ-12, CD-RS, MSPSS, UCLA-8, ISI, IES-6 and DASS-21. We identified two main types of stressors in our population. Being diagnosed with a pregnancy complication, having an ectopic pregnancy or a spontaneous abortion were identified as pregnancy-related stressors. On the other hand, infections or deaths among partners or relatives were defined as covid-related stressors. Scale scores were compared considering main sociodemographic data and possible stressors, both pregnancy-related and covid-related, that may affect women’s mental health. Fisher exact test, Chi square Test and Student’s T test were used when appropriate.

**Results:**

Among 98 women with complete data at baseline, 21,3% suffered from at least mild anxiety, 21% suffered from at least mild depression, and 41% experienced at least mild stress according to the DASS-21 scale. Moreover, 26,4% reported PTSD symptoms according to the IES-6 scale. Women who experienced a stressor were more likely to report psychiatric symptoms, even more so when exposed to a pregnancy-related stressor. Being Caucasian, a personal psychiatric history or a familial psychiatric history correlated with scoring positive to a higher number of scales. Regarding DASS-21, high total scores in this scale correlated with being Caucasian and having had previous deliveries. More specifically, being Caucasian, older age, previous deliveries and a positive psychiatric history correlated with a higher score in the depression subscale. A positive psychiatric history and a familial psychiatric history correlated with higher scores in the anxiety subscale, while being hospitalized at a late gestational age correlated with lower scores in this section. Scoring above the cut-off of the DASS stress subscale correlated with a positive psychiatric history, a positive familial psychiatric history, being Caucasian, and with an older age of the women in the sample.

**Conclusions:**

Our results underline the burden of the COVID-19 pandemic on women in pregnancy and in the perinatal period. Tailored interventions should be staged to provide adequate care to this particularly sensitive population, for the wellbeing of both the mother and the child.

**Disclosure of Interest:**

None Declared

